# Supplementary material for: COVID-19 vaccination intention among internally displaced persons in complex humanitarian emergency context, Northeast Nigeria
Source: PLoS One. 2024 Aug 30;19(8):e0308139. doi: 10.1371/journal.pone.0308139 (PMC11364247; doi:10.1371/journal.pone.0308139)
Supplement: S1 Checklist — (DOCX) [file pone.0308139.s001.docx]

Inclusivity in global research

PLOS’ policy on inclusivity in global research aims to improve transparency in the reporting of research performed outside of researchers’ own country or community and ensures that PLOS publications reporting global research adhere to high standards for research ethics and authorship. Authors of relevant research articles may be asked to complete the questionnaire below, which outlines ethical, cultural, and scientific considerations specific to inclusivity in global research. This questionnaire may be requested when researchers have travelled to a different country to conduct research, if research uses samples collected in another country, research with Indigenous populations or their lands, or if research is on cultural artefacts. Researchers travelling to another country solely to use laboratory equipment will not normally be required to complete the questionnaire. However, the questionnaire can be requested at the journal’s discretion for any submission – if you have been requested to complete this questionnaire by the PLOS journal you submitted to, please do so.

Please complete the questionnaire below and include this as a Supporting Information file with your manuscript. Note that if your paper is accepted for publication, this checklist will be published with your article in the supporting information files. Please ensure that you reference the checklist in the main body of your manuscript. We suggest adding a subsection ‘Inclusivity in global research’ to your Methods section and adding the following sentence: “Additional information regarding the ethical, cultural, and scientific considerations specific to inclusivity in global research is included in the Supporting Information (SX Checklist)”

The questions have been designed to be applicable to a wide range of study types, and there are subsections for both human subjects research and non-human subjects research. If any of the questions are not relevant to your research please mark them as “N/A” as appropriate.

**Ethical considerations, permits and authorship**

*This section is applicable to all research types.*

Provide details as to who granted permissions and/or consent for the study to take place in the Methods section of your manuscript. This should include the names of **all** ethics boards, governmental organizations, community leaders or other bodies that provided approval for the study. If individuals provided approval refer to these people by their role or title but do not list their name(s).

Reported on pages number: **223 – 229**

If there were any deviations from the study protocol after approval was obtained please provide details of these changes in the Methods section of your manuscript.
Did this study involve local collaborators that are residents of the country where the research was conducted or members of the community studied? If you do not have any authors from said communities, please provide an explanation for this below.

Reported on page number: **None**

**Yes,**

**Local collaborators from the communities participated in the research. Those who met the PLOS criteria for authorship are listed as authors in the study and are included in the author byline.**

Everyone listed as an author should meet PLOS’ criteria for authorship and all individuals who meet these criteria should be included in the author byline, rather than the acknowledgements. For further information please see the journal’s Authorship Policy.

**Human subjects research (e.g. health research, medical research, cross-cultural psychology)**

Did you obtain written informed consent from a representative of the local community or region before the research took place? How did you establish who speaks for the community? Details of written informed consent obtained from study participants should be reported separately in the Methods section of your manuscript.

**Yes, we obtained written consent, permission and approval from appropriate local authorities before the research. Our participants were internally displaced persons (IDPs) residing in IDPs camps in three States in northeast Nigeria. The Ministries of Health in these States, and the Emergency Management Agencies, provided the lead to the representatives of the study IDPs across the States.**

How did members of the local community provide input on the aims of the research investigation, its methodology, and its anticipated outcome(s)?

**Representatives of the local communities were briefed about the research, including the benefits and expected outcomes before entry into the IDP camps for data collection. Collaborators from the local environment participated in the identification of research assistants and data collectors from among the community members. They also participated in the pilot study and provided substantial input during the revision of the protocols and questionnaires post-pilot. Given the enormous insecurity in the research areas, members of the local communities served as local guides and provided local security for the research assistants during data collection.**

When engaging with the local community, how did you ensure that the informed consent documents and other materials could be understood by local stakeholders?

**Prior to the conduct of the research, a standard informed consent was developed and submitted to the National Health Research Ethics Committee of Nigeria (NHREC). The informed consent, developed in English Language, was reviewed and subsequently approved by NHREC. To facilitate understanding, the informed consent was translated into Hausa – the local language and explained in detail to local stakeholders. The benefit of the research was also explained in local language. Voluntary participation and opportunity to withdraw at any stage of the research without prejudice was emphasized. The States’ Ministries of Health (of Borno, Adamawa and Yobe) who granted approval and permission to conduct the study in the IDPs camps provided strategic oversight and supervison during the research period ensuring that all aspects of the informed consent as presented were adhered to. The privacy of the respondents was respected. For security reasons and fear of being persecuted, the identities of those who participated in the research were hidden.**

Will the findings of the research be made available in an understandable format to stakeholders in the community where the study was conducted (e.g. via a presentation, summary report, copies of publications, etc.)? Please provide details of how this will be achieved.

**Yes, the findings and recommendations of the research will be made available to local stakeholders including the States’ Ministries of Health of Borno, Adamawa and Yobe States, and the leadership of the IDPs camps. Once the research is published, we will make copies of the publications available to the States’ Ministries of Health, States’ Emergency Management Agency and other local stakeholders.**

**Non-human subjects research using specimens/ animals collected as part of the study, or those housed in archival collections. Examples include archaeology, paleontology, botany and zoology.**

Did the permission you obtained from a local authority to perform the study include an agreement on access to outputs and benefit sharing? This may include procedures to enable fair distribution of the benefits and resources arising from the research performed. Please include any details of Prior Informed Consent and Benefit Sharing Agreements obtained. These may be required by field-specific regulations, for example the Convention on Biological Diversity (CBD) and the associated Nagoya Protocol.

**N/A**

If the material used in your study was imported, please A) provide the year it was imported and B) indicate whether permits were obtained to import/export the materials used, C) provide details of any permits obtained. If this information is not available, please indicate this.

**N/A**

If you used archival specimens, please state how the material used in your study was acquired by the institute it is held in and provide details of any permits obtained for the original excavations/ sample collection. If this information is not available, please indicate this.

**N/A**

How was the potential cultural significance of the materials collected in your study to local communities considered in your research design? Were Indigenous peoples and/or local researchers and institutions involved with archaeological excavations / collection of specimens? If so, please provide a description of their involvement.

**N/A**

If your manuscript includes photographs of human remains please indicate whether authors obtained permission from descendants or affiliated cultural communities to do so.

**N/A**
