# Supplementary material for: COVID-19 vaccination intention among internally displaced persons in complex humanitarian emergency context, Northeast Nigeria
Source: PLoS One. 2024 Aug 30;19(8):e0308139. doi: 10.1371/journal.pone.0308139 (PMC11364247; doi:10.1371/journal.pone.0308139)
Supplement: S1 File — (PDF) [file pone.0308139.s002.pdf]

# Data tool for COVID-19 Study

DATA TOOL FOR COVID-19 STUDY

DO YOU CONSENT TO PARTICIPATE IN THE STUDY? *

- (●) Yes
- ( ) No

**1. NAME OF INTERVIEWER** *

*Sunan Mai yin tambaya*

---

**2. DATE OF INTERVIEW** *

*Ranan da ake yin tanmabaya*

yyyy-mm-dd

---

**3. STATE:** *

*Jaha*

- ( ) Adamawa
- (x) Borno
- ( ) Yobe

---

**4. LGA (OF IDPS CAMP LOCATION)** *

*K'aramar hukumar Jaha*

- ( ) Abadam
- (x) Askira/Uba
- ( ) Bama
- ( ) Bayo
- ( ) Biu
- ( ) Chibok
- ( ) Damboa
- ( ) Dikwa
- ( ) Gubio
- ( ) Guzamala
- ( ) Gwoza
- ( ) Hawul
- ( ) Jere
- ( ) Kaga
- ( ) Kala/Balge

---

**5. WARD (OF IDPS CAMP LOCATION)** *

*Karamar mazaba*

- (x) Askira East
- ( ) Dille/Huyim
- ( ) Hausari Zadawa
- ( ) Hussara/Tampul
- ( ) Lassa
- ( ) Mussa
- ( ) Ngohi
- ( ) Ngulde
- ( ) Ngurthlafu/Kopa
- ( ) [illegible]
- ( ) Uba
- ( ) Uvu Uda
- ( ) Wamdeo

## IDP CAMP CHARACTERISTICS

---

**6. NAME OF IDPS CAMP** *

*Sunan IDP camp*

---

**7. NAME OF SETTLEMENT IDPS CAMP IS LOCATED** *

*Sunan Anguwar da wanan IDP Camp ke chiki*

8. STATUS OF IDPS CAMP *

*Matakin IDP camp*

- ○ Formal
- ○ Informal

9. WHAT YEAR WAS THE IDPS CAMP ESTABLISHED? *

*A wace shekara a bud'e wannan IDP camp?*

yyyy

10. IS THERE ANY HEALTH FACILITY IN THE IDPS CAMP? *

*Akwai asibiti ko dakin kiwon lafiya a wannan camp?*

- ○ Yes
- ○ No

11. IF YES, HOW MANY HEALTH FACILITIES ARE IN THIS IDPS CAMP? *

*Idan akwai, Asbitoci/Dakunan kiwon lafiya guda nawa ne a wannan Kamp (Camp) din?*

12. APART FROM TREATMENT OF COMMON AILMENTS, WHAT OTHER SERVICES ARE PROVIDED BY THE HEALTH FACILITY(IES)? MULTIPLE RESPONSES ALLOWED. CHOOSE ALL THAT APPLIES. *

(CHOOSE ALL THAT APPLIES)

*Bayan samarda kula ga cutuka na yau da kullum, wa'yanne kula ake samarwa a wannan/ wa'yannan asbitocin ko dakunan kiwon lafiya?*

*Multiple responses are allowed*

- ☐ Nutrition
- ☐ Ante-natal care (ANC)
- ☐ Routine Immunization (RI)
- ☐ Family Planning
- ☐ No other services
- ☐ Others (please specify)

OTHERS, PLEASE SPECIFY

13. GEOGRAPHICAL STRATUM/DIVISION OF THE CAMP (BASED ON CAMPAIGN MICROPLANNING) *

- ○ 1
- ○ 2
- ○ 3
- ○ 4

## SOCIO-DEMOGRAPHIC CHARACTERISTICS OF RESPONDENT

14. NAME OF RESPONDENT: (FIRST NAME, LAST NAME) *

*Sunan mai bada ansa (ko Sunan wanda ke baiwa marar lafiya kula)*

15. SEX *

*Jinsin mai bada kula (mache ko na miji)*

- ◯ Male
- ◯ Female

16. AGE IN YEARS (AS AT LAST BIRTHDAY) *

*Shekarun mai bada ansa (ko mai bada kula)*

17. HIGHEST LEVEL OF FORMAL EDUCATION ATTAINED *

*Matakin ilimi/ karatu*

- ◯ None
- ◯ Primary
- ◯ Secondary
- ◯ Tertiary

18. PRESENT MARITAL STATUS *

*Matsayin auren mai bada amsa (ko mai baiwa marar lafiya kula)*

- ◯ Never married
- ◯ Presently married
- ◯ Presently seperated
- ◯ Presently divorced
- ◯ Widowed

19. OCCUPATION *

*Sana'a*

- ◯ Unemployed
- ◯ Farmer
- ◯ Housewife
- ◯ Student
- ◯ Artisan
- ◯ Unskilled labourer
- ◯ Skilled labourer
- ◯ Private job
- ◯ Government Job
- ◯ Business
- ◯ Healthcare worker
- ◯ Others (specify)

OTHERS, PLEASE SPECIFY

20. RELIGION *

*Addini*

- ◯ Islam
- ◯ Christianity
- ◯ Traditional
- ◯ Others (please specify)

OTHERS, PLEASE SPECIFY

21. STATE OF HABITUAL RESIDENCE (BEFORE DISPLACEMENT) *

*Jahar tushe (Kafin hijira)*

- ◯ Adamawa
- ◯ Bauchi
- ◯ Benue
- ◯ Borno
- ◯ FCT, Abuja
- ◯ Gombe
- ◯ Jigawa
- ◯ Kaduna
- ◯ Kano
- ◯ Katsina
- ◯ Kebbi
- ◯ Kogi
- ◯ Kwara
- ◯ Nasarawa
- ◯ Niger
- ◯ Plateau
- ◯ Sokoto
- ◯ Taraba
- ◯ Yobe
- ◯ Zamfara

22. LGA OF HABITUAL RESIDENCE (BEFORE DISPLACEMENT) *

*K'aramar hukuman tushe (kafin hijira)*

23. FOR HOW LONG HAVE YOU BEEN LIVING IN THIS CAMP (IN YEARS)? *

*Kunyi Kimanin Shekaru nawa a wannan IDP Camp?*

- ◯ Less than 1 year
- ◯ 1 year to less than 2 years
- ◯ 2 years to less than 5 years
- ◯ 5 years and above

24. HOW MANY PEOPLE LIVE IN YOUR HOUSEHOLD, INCLUDING YOU? *

*Mutane nawa ke zama a wannan muhalli tare kai/ke?*

..............................................................................................................................

25. WHAT IS THE AVERAGE MONTHLY INCOME OF YOUR HOUSEHOLD (IN NAIRA)? *

*Kimanta kudin da Mazauna wannan Gida suke samu a wata (Naira)?*

..............................................................................................................................

26. WHAT IS THE DISTANCE FROM WHERE YOU LIVE TO THE NEAREST HEALTH FACILITY? (IN KILOMETERS) *

*Me nene Nisa/tazara tsakanin muhalli da wurin samun kiwon lafiya (Kilo mita nawa)*

- ◯ Less than 1km
- ◯ Between 1 and 2km
- ◯ Between 2 and 3km
- ◯ Between 3 and 5km
- ◯ Between 5 and 10km
- ◯ More than 10km

KNOWLEDGE OF COVID-19

27. HAVE YOU HEARD OF CORONAVIRUS DISEASE (COVID-19)? *

*Ka/kin taba samun labarin Korona (COVID19)?*

- ◯ Yes
- ◯ No

28. WHAT CAUSES CORONAVIRUS DISEASE (COVID-19)? *

(MULTIPLE RESPONSES ALLOWED. INTERVIEWERS DO NOT READ OPTIONS)

*Me ke Kawo cutar Korona????*

*Multiple responses are allowed*

- ☐ Germ (microbe)
- ☐ Spiritual attack
- ☐ Jinn
- ☐ Witchcraft
- ☐ Mosquito
- ☐ Don't know
- ☐ Others (please specify)

OTHERS, PLEASE SPECIFY

*

29. WHAT ARE THE SIGNS AND SYMPTOMS OF CORONAVIRUS DISEASE (COVID-19)?

(MULTIPLE RESPONSES ALLOWED. INTERVIEWERS DO NOT READ OPTIONS)

*Menene alamun cutar Korona?*

*Multiple responses are allowed*

- [ ] Fever
- [ ] Headache
- [ ] Rash
- [ ] Cough
- [ ] Catarrh
- [ ] Difficulty in breathing
- [ ] Neck stiffness
- [ ] Tiredness
- [ ] Nausea
- [ ] Loss of smell
- [ ] Loss of taste
- [ ] Joint pain
- [ ] Chest pain
- [ ] Vomiting
- [ ] Conjunctivitis
- [ ] Diarrhoea
- [ ] No signs and symptoms in some cases
- [ ] Don't know
- [ ] Others (specify)

OTHERS, PLEASE SPECIFY

30. HOW CAN A PERSON CONTRACT (GET) CORONAVIRUS DISEASE (COVID-19)? *

(MULTIPLE RESPONSES ALLOWED. INTERVIEWERS DO NOT READ OPTIONS)

*Ya mutum ke kamuwa da cutar Korona?*

*Multiple responses are allowed*

- [ ] Mosquito bite
- [ ] Drinking polluted water
- [ ] Through sexual intercourse
- [ ] Touching surfaces or objects in a public place
- [ ] Through shaking hand
- [ ] Through contact with animals
- [ ] Through hugging people
- [ ] Through contact with a person who is sick of COVID-19
- [ ] By touching body fluid of a person who is sick with COVID-19
- [ ] Through spiritual attack
- [ ] Through insect bite
- [ ] From a blood transfusion
- [ ] Don't know
- [ ] Other (please specify)

OTHERS, PLEASE SPECIFY

31. DOES EVERYBODY WHO GETS CORONAVIRUS (COVID-19) SHOW SIGNS AND SYMPTOMS? *

*Duk Wanda Ya kamu da chutar Korona ne Yake nuna alamun Cutan?*

- ○ Yes
- ○ No
- ○ Don't know

32. CAN CORONAVIRUS DISEASE (COVID-19) BE PREVENTED? *

*Ana iya daukan mataken kare kamuwa da cuntar Korona?*

- ○ Yes
- ○ No
- ○ Don't know

*

33. IF YES, HOW CAN YOU PREVENT YOURSELF FROM CONTRACTING (GETTING) CORONAVIRUS DISEASE (COVID-19)

(MULTIPLE RESPONSES ALLOWED. INTERVIEWERS DO NOT READ OPTIONS)

*Idan ana iyawa, ta ya za Ka/ki iya kare kai daga samun cutar Korona?*

- [ ] Avoid shaking hands while greeting
- [ ] Covering mouth and nose properly when coughing or sneezing
- [ ] Washing hands properly after touching items in public places
- [ ] Avoid overcrowded places
- [ ] Using mosquito repellent or spray on your body
- [ ] Wear full body covering clothes
- [ ] Covering mouth with a mask
- [ ] Abstain from sexual intercourse
- [ ] Keeping household and environment clean
- [ ] Using mosquito net
- [ ] Using hand sanitizers
- [ ] Through good nutrition
- [ ] By praying
- [ ] By taking COVID-19 vaccine
- [ ] Don't know
- [ ] Others (please specify)......

OTHERS, PLEASE SPECIFY

..........................................................................................

34.IS THERE A TREATMENT FOR CORONAVIRUS DISEASE (COVID-19)? *

*Akwai maganin warkarwa daga chutar Korona?*

- ○ Yes
- ○ No
- ○ Not sure
- ○ Don't know

35.IF YES, WHAT IS THE TREATMENT? *

*Idan akwai, Menene wannan magani*

..........................................................................................

36. IS THERE IS A VACCINE TO PREVENT CORONAVIRUS DISEASE (COVID-19)? *

*Akwai rigakafin cutar Korona?*

- ◯ Yes
- ◯ No
- ◯ Not sure
- ◯ Don't know

37. WHAT IS YOUR SOURCE OF INFORMATION ABOUT CORONAVIRUS DISEASE (COVID-19)? *

(MULTIPLE RESPONSES ALLOWED. INTERVIEWERS DO NOT READ OPTIONS)

*Menene hanyar da Kake/Kike samu bayane akan chutar Korona?*

*Multiple responses are allowed*

- ☐ Radio
- ☐ Television
- ☐ Health worker
- ☐ Community informant
- ☐ Polio teams
- ☐ Social media
- ☐ Newspapers
- ☐ Posters
- ☐ Religious centres
- ☐ Schools
- ☐ Health facility
- ☐ Market
- ☐ Others (specify)

OTHERS, PLEASE SPECIFY

COVID-19 RISK PERCEPTION

NOW, I AM GOING TO READ SOME STATEMENTS, I WOULD LIKE YOU TO TELL ME HOW WELL YOU AGREE OR DISAGREE WITH EACH STATEMENT:

*Yanzu zan karanto wasu ayoyi, Inaso Ka/Ki gayamin yanayin gamsuwa ko rashin gamsuwa da kayi/Ki kayi dasu:*

PERCEIVED THREAT: SEVERITY

38. CORONAVIRUS DISEASE (COVID-19) IS A SEVERE DISEASE *

*Korona chuta che mai matuk'an tsanani*

- ◯ Strongly Disagree
- ◯ Disagree
- ◯ Neutral (Neither agree or disagree)
- ◯ Agree
- ◯ Strongly Agree

39. CORONAVIRUS DISEASE (COVID-19) CAN HAVE SERIOUS CONSEQUENCES ON MY LIFE AND LIVELIHOOD *

*Korona cuta ce dake iya kawo tangarda a rayuwa na da kuma hanyar samu na*

- ◯ Strongly Disagree
- ◯ Disagree
- ◯ Neutral (Neither agree or disagree)
- ◯ Agree
- ◯ Strongly Agree

40. CORONAVIRUS DISEASE (COVID-19) IS VERY HARMFUL *

*Cutar Korona tana illa sosai matuqa.*

- ◯ Strongly Disagree
- ◯ Disagree
- ◯ Neutral (Neither agree or disagree)
- ◯ Agree
- ◯ Strongly Agree

PERCEIVED THREAT: SUSCEPTIBILITY

41. I AM AT RISK OF GETTING CORONAVIRUS DISEASE (COVID-19) *

*Akwai hatsarin na kamu da cutar Korona*

- ◯ Strongly Disagree
- ◯ Disagree
- ◯ Neutral (Neither agree or disagree)
- ◯ Agree
- ◯ Strongly Agree

42. IT IS LIKELY (OR POSSIBLE) THAT I WILL GET CORONAVIRUS DISEASE (COVID-19) *

*Akwai yiwuwar na kamu da cutar Korono*

- Strongly Disagree
- Disagree
- Neutral (Neither agree or disagree)
- Agree
- Strongly Agree

43. I AM SUSCEPTIBLE TO CORONAVIRUS DISEASE (COVID-19) *

*Ina iya kamuwa da cutar Korona*

- Strongly Disagree
- Disagree
- Neutral (Neither agree or disagree)
- Agree
- Strongly Agree

PERCEIVED EFFICACY: RESPONSE EFFICACY

RESPONSE EFFICACY: PHYSICAL DISTANCING

44A. MAINTANING PHYSICAL DISTANCING IS EFFECTIVE IN PREVENTING COVID-19 *

*Barin tazara tsakanin mutane yana iya zama kariya daga Cutar Korona*

- Strongly Disagree
- Disagree
- Neutral (Neither agree or disagree)
- Agree
- Strongly Agree

44B. AVOIDING CROWDED PLACES REDUCES THE RISK OF CONTRACTING COVID-19 *

*Nisantar Taro/chinkoso na rage yiwuwar kamuwa da korona*

- Strongly Disagree
- Disagree
- Neutral (Neither agree or disagree)
- Agree
- Strongly Agree

44C. IF I PRACTICE PHYSICAL DISTANCING, I AM LESS LIKELY TO GET COVID-19 *

*Idan na kiyaye umurnin bada tazara wiwuwan kamuwana zai ragu*

- Strongly Disagree
- Disagree
- Neutral (Neither agree or disagree)
- Agree
- Strongly Agree

RESPONSE EFFICACY: FACE MASK

45A. FACE MASK IS EFFECTIVE IN PREVENTING COVID-19 *

*Takunkumin fiska yana bada kariya daga korona*

- Strongly Disagree
- Disagree
- Neutral (Neither agree or disagree)
- Agree
- Strongly Agree

45B. WEARING FACE MASK REGULARLY PROTECTS AGAINST COVID-19 *

*Sa Takunkumin akai akai na bada kariya daga cutar korona*

- Strongly Disagree
- Disagree
- Neutral (Neither agree or disagree)
- Agree
- Strongly Agree

45C. IF I WEAR FACE MASK REGULARLY, I AM LESS LIKELY TO CONTRACT COVID-19 *

*idan na sa takunkumi akai akai yiwuwar kamuwa na zai ragu.*

- Strongly Disagree
- Disagree
- Neutral (Neither agree or disagree)
- Agree
- Strongly Agree

RESPONSE EFFICACY: HYGIENE MEASURES

46A. CLEANING HANDS REGULARLY WITH SOAP AND WATER OR HAND SANITIZER IS EFFECTIVE IN PREVENTING COVID-19 *

*Wanke hannaye akan kari da ruwa da saubulu ko (man wanke hanu) Sanitiza ya da tasiri wurin bada kariya daga cutar korona*

- Strongly Disagree
- Disagree
- Neutral (Neither agree or disagree)
- Agree
- Strongly Agree

46B. CLEANING HANDS REGULARLY WITH SOAP AND WATER OR HAND SANITIZER DETERS COVID-19 *

*Wanke hannaye akan kari da ruwa da saubulu ko (man wanke hanu) Sanitiza ya na kawar da Korona*

- Strongly Disagree
- Disagree
- Neutral (Neither agree or disagree)
- Agree
- Strongly Agree

46C. I CAN REDUCE THE RISK OF CONTRACTING COVID-19 BY CLEANING MY HANDS REGULARLY WITH SOAP AND WATER OR HAND SANITIZER *

*Ina Iya rage yiwuwar kamuwa na da cutar korona idan ina wanke hannaye akan kari da ruwa da saubulu ko (man wanke hanu) Sanitiza ko dayaushe.*

- Strongly Disagree
- Disagree
- Neutral (Neither agree or disagree)
- Agree
- Strongly Agree

RESPONSE EFFICACY: COVID-19 VACCINATION

47A. COVID-19 VACCINE PROTECTS AGAINST SERIOUS ILLNESS AND DEATH FROM COVID-19 *

*Rigakafin chutan korona na bada kariya daga jinya mai tsanani ko mutuwa daga cutar korona*

- Strongly Disagree
- Disagree
- Neutral (Neither agree or disagree)
- Agree
- Strongly Agree

47B. COVID-19 VACCINE IS EFFECTIVE IN PREVENTING SERIOUS COVID-19 DISEASE OR COVID-19 DEATH *

*Rigakafin cutar korona yana da tasiri wurin bada kariya daga cutar koronar mai tsanani ko mutuwa ta hanyan korona*

- Strongly Disagree
- Disagree
- Neutral (Neither agree or disagree)
- Agree
- Strongly Agree

47C. IF I AM VACCINATED WITH COVID-19 VACCINE, I WILL BE PROTECTED FROM SERIOUS COVID-19 DISEASE OR COVID-19 DEATH *

*Idan na dauki Rigakafin cutar korona zan samu kariya daga cutar korona mai tsanani ko mutuwa ta hanyan korona.*

- Strongly Disagree
- Disagree
- Neutral (Neither agree or disagree)
- Agree
- Strongly Agree

PERCEIVED EFFICACY: SELF-EFFICACY

SELF-EFFICACY: PHYSICAL DISTANCING

48A. IT IS EASY FOR ME TO MAINTAIN PHYSICAL DISTANCING TO PREVENT COVID-19 *

*Barin tazara tsakani na da mutane, abune mai sauqi agareni domin samun kariya da cutar Korona*

- Strongly Disagree
- Disagree
- Neutral (Neither agree or disagree)
- Agree
- Strongly Agree

48B. I CAN AVOID CROWDED PLACES TO REDUCE MY RISK OF CONTRACTING COVID-19 *

*Zan iya qaurache wa taro/cinkoso domin rage hatsarin kamuwa na da cutar Korona*

- Strongly Disagree
- Disagree
- Neutral (Neither agree or disagree)
- Agree
- Strongly Agree

48C. I AM ABLE TO PRACTICE PHYSICAL DISTANCING TO PREVENT COVID-19 *

*Ina iya kaddamadda barin tazara domin kwaurachewa taro/chinkoso domin samun karita daga kama chutar Korona*

- Strongly Disagree
- Disagree
- Neutral (Neither agree or disagree)
- Agree
- Strongly Agree

SELF-EFFICACY: FACE MASK

49A. I AM ABLE TO WEAR FACE MASK REGULARLY TO PREVENT COVID-19 *

*Ina da daman sa takunkumi a koyaushe domin samun kariya daga chutar Korona*

- Strongly Disagree
- Disagree
- Neutral (Neither agree or disagree)
- Agree
- Strongly Agree

49B. IT IS EASY FOR ME TO WEAR FACE MASK REGULARLY TO PREVENT COVID-19 *

*Saka Takunkumi koda yaushe domin samun kariya daga cutar korona, abune mai sauqi agareni.*

- Strongly Disagree
- Disagree
- Neutral (Neither agree or disagree)
- Agree
- Strongly Agree

49C. I CAN WEAR FACE MASK REGULARLY TO PREVENT COVID-19 *

*Zan iya sanya takunkumi koda yaushe domin samun kariya daga chutar Korona*

- Strongly Disagree
- Disagree
- Neutral (Neither agree or disagree)
- Agree
- Strongly Agree

SELF-EFFICACY: HYGIENE MEASURES

50A. I AM ABLE TO CLEAN MY HANDS REGULARLY WITH SOAP AND WATER OR HAND SANITIZER TO PREVENT COVID-19 *

*Ina da daman tsabtache hannaye na da ruwa da sabulu ko man wanke hanu(Sanitiza) domin samon kariya daga cutar Korona*

- ◯ Strongly Disagree
- ◯ Disagree
- ◯ Neutral (Neither agree or disagree)
- ◯ Agree
- ◯ Strongly Agree

50B. IT IS CONVENIENT FOR ME TO CLEAN MY HANDS REGULARLY WITH SOAP AND WATER OR HAND SANITIZER TO PREVENT COVID-19 *

*Tsabtache hannaye na daruwa da sabubu ko man wanke hanu(Sanitiza) domin samun kariya daga chutar Korona yana da sauki agare ni.*

- ◯ Strongly Disagree
- ◯ Disagree
- ◯ Neutral (Neither agree or disagree)
- ◯ Agree
- ◯ Strongly Agree

50C. I CAN CLEAN MY HANDS REGULARLY WITH SOAP AND WATER OR HAND SANITIZER TO PREVENT COVID-19 *

*Zan iya tsabtache hannaye na da ruwa da sabulu ko man wanke hannu(Sanitiza) domin samun kariya daga cutar Korona*

- ◯ Strongly Disagree
- ◯ Disagree
- ◯ Neutral (Neither agree or disagree)
- ◯ Agree
- ◯ Strongly Agree

SELF-EFFICACY: COVID-19 VACCINATION

51A. I CAN TAKE COVID-19 VACCINE TO PROTECT ME FROM SERIOUS ILLNESS OR DEATH FROM COVID-19 DSEASE? *

*Zan iya daukan rigakafin cutar Korona domin kare kaina daga jinya mai tsanani ko mutuwa ta hanyar cutar korona*

- ◯ Strongly Disagree
- ◯ Disagree
- ◯ Neutral (Neither agree or disagree)
- ◯ Agree
- ◯ Strongly Agree

51B. I AM ABLE TO RECEIVE COVID-19 VACCINE TO PREVENT SERIOUS ILLNESS OR DEATH FROM COVID-19 DISEASE *

*Ina da daman karb'an rigakafin cutar Korona domin samun kariya daga jinya mai tsanani ko mutuwa ta hanyar cutar korona*

- Strongly Disagree
- Disagree
- Neutral (Neither agree or disagree)
- Agree
- Strongly Agree

51C. IT IS EASY FOR ME TO TAKE COVID-19 VACCINE TO PREVENT SERIOUS ILLNESS OR DEATH FROM COVID-19 DISEASE *

*karb'an rigakafin cutar Korona domin samun kariya da jinya mai tsanani ko mutuwa daga cutar korona yana da sauki*

- Strongly Disagree
- Disagree
- Neutral (Neither agree or disagree)
- Agree
- Strongly Agree

PRACTICES REGARDING COVID-19

52. SINCE THE CORONAVIRUS DISEASE (COVID-19) PANDEMIC STARTED, HAVE YOU TAKEN ANY ACTION(S) OR MEASURES TO PREVENT YOURSELF AND YOUR HOUSEHOLD MEMBERS FROM GETTING THE DIEASE? *

*Tun shigowan annoban Korona ka dauki wani matakai domin kare kanka ko iyali ka daga kamuwa daga wannan cutar?*

- Yes
- No

*

53. IF YES, WHAT ACTION(S) OR MEASURES HAVE YOU TAKEN

(MULTIPLE RESPONSES ALLOWED. INTERVIEWERS DO NOT READ OPTIONS)

*Idan ka dauki matakai, wa danne matakai ka/kika dauka?*

*Multiple responses are allowed*

- [ ] Using mask
- [ ] Sprayed or fumigated my home
- [ ] Washed hand for at least 20 seconds daily with soap
- [ ] Used sanitizer
- [ ] COVID-19 vaccination
- [ ] Avoided handshake and hugging
- [ ] Avoided touching objects at public places
- [ ] Avoided going outside of the home
- [ ] Prayed to God
- [ ] No action is taken as I am not at risk
- [ ] Other (please mention)

OTHERS, PLEASE SPECIFY

*

54. IF NO, WHY HAVEN'T YOU TAKEN ANY ACTION?

(MULTIPLE RESPONSES ALLOWED. INTERVIEWERS DO NOT READ OPTIONS)

*Idan Ba ka dauki mataki ba, menene dalilin?*

*Multiple responses are allowed*

- [ ] I am not at risk / my household is not at risk
- [ ] Coronavirus disease (Covid-19) is not a problem
- [ ] Coronavirus diseases is not real
- [ ] I do not have the resources or access to preventative measures
- [ ] I do not think preventive measures are effective
- [ ] I do not have access to information on how to prevent myself and household
- [ ] Others (specify)

OTHERS, PLEASE SPECIFY

55. WHAT WOULD YOU DO IF YOU CONTRACT (GET) COVID-19 *

(MULTIPLE RESPONSES ALLOWED. INTERVIEWERS DO NOT READ OPTIONS)

*Wane mataki zaka/Zaki dauka idan ka/ki ka kamu daga cutar Korona?*

*Multiple responses are allowed*

- [ ] Do nothing
- [ ] Go to health facility
- [ ] Self medication
- [ ] Go to traditonal healer
- [ ] Call a health worker
- [ ] Go to religious centre
- [ ] Pray
- [ ] Isolate myself
- [ ] Keep my family away
- [ ] Others (specify)

OTHERS, PLEASE SPECIFY

UPTAKE OF COVID-19 VACCINE

56. HAVE YOU RECEIVED CORONAVIRUS DISEASE (COVID-19) VACCINE? *

*Ka/Kin karbi rigakafin cutar Korona?*

- ○ Yes
- ○ No

57. HOW MANY DOSES HAVE YOU RECEIVED? *

*Har Allurai nawa ka/kika karb'a?*

- ○ One dose
- ○ Two doses
- ○ Two doses PLUS one booster dose
- ○ Two doses PLUS two booster doses

58. WHY DID YOU TAKE ONLY ONE DOSE? (FOR RESPONDENTS WHO RECEIVE ONLY ONE DOSE) *

(MULTIPLE RESPONSES ALLOWED. INTERVIEWERS DO NOT READ OPTIONS)

*Meye dalilin ka/ki na karb'an daya kawai?*

*Multiple responses are allowed*

- [ ] Don't know that I am supposed to take more than one dose
- [ ] Because of side effects of the first dose
- [ ] No felt need
- [ ] I thought only one dose is adequate
- [ ] Vaccine was not available again?
- [ ] Diffcult to access where I can receive vaccine
- [ ] COVID-19 vaccine is not safe
- [ ] No consent from husband/household head
- [ ] Vaccination teams are selling the vaccine, and I don't have money to pay
- [ ] Poor attitude of health workers
- [ ] I don't have any reason
- [ ] Others, (please specify)

OTHERS, PLEASE SPECIFY

................................................................................

59. WHERE DID YOU RECEIVE THE (LAST OR THE ONLY) DOSE OF COVID-19 VACCINE? *

*Ina ka/ki karbi alluran karshe na rigakafin cutar korona?*

- ○ Health facility in the camp
- ○ Health facility outside the camp
- ○ COVID-19 vaccination outreach/post in the camp
- ○ COVID-19 vaccination outreach/post outside the camp
- ○ Others, (please specify)

OTHERS, PLEASE SPECIFY

................................................................................

60. DO YOU HAVE A VACCINATION CARD FOR CORONAVIRUS DISEASE (COVID-19) VACCINE? *

*Ka/ki nada kattin shaida na alluran rigakafin cutar korona?*

- ○ Yes
- ○ No

*

61. IF YES, CHECK THE CARD, AND INDICATE:

(MULTIPLE RESPONSES ALLOWED)

*Idan A ne, Toh duba Katin Shaida, Sai kuma a rubuta;*

*Multiple responses are allowed*

- [ ] First dose recorded on the card
- [ ] Second dose recorded on the card
- [ ] First booster dose recorded on the card
- [ ] Second booster dose recorded on the card

62. WHY DON'T YOU HAVE A VACCINATION CARD? *

*Idan A'a ne, A tambaya dalili*

- ○ Vaccination card exhausted and not given
- ○ Vaccination team said card not neccesary and not given
- ○ Vaccination team demanded for money to give card
- ○ No felt need
- ○ Vaccination card lost
- ○ Others (please specify)

OTHERS, PLEASE SPECIFY

........................................................................................................

*

63. WHY HAVEN'T YOU RECEIVE COVID-19 VACCINE?

(MULTIPLE RESPONSES ALLOWED. INTERVIEWERS DO NOT READ OPTIONS)

*Meye dalilin rashin karban rigakafin Cutar Korona?*

*Multiple responses are allowed*

- [ ] COVID-19 is a deception, it does not exist
- [ ] I don't belief in COVID-19
- [ ] No felt need
- [ ] COVID-19 vaccine is not safe
- [ ] COVID-19 vaccine can cause infertility
- [ ] Vaccine not available at health facility
- [ ] Far distance from vaccination site
- [ ] Vaccination teams are selling the vaccine, and I don't have money to pay
- [ ] Fear of vaccine side effects
- [ ] No consent from husband/household head
- [ ] Poor attitude of health workers
- [ ] I have not heard of COVID-19 vaccine before
- [ ] I don't have any reason
- [ ] Others (please specify)

OTHERS, PLEASE SPECIFY

..............................................................................................................................................

64. WILL YOU RECEIVE COVID-19 VACCINE IN NEAREST FUTURE? *

*Za ka/ki karb'l alluran rigakafin a kwana kusa?*

- ( ) Yes
- ( ) No

65. IF NO, WHY? *

*idan A'a meye dalili?*

..............................................................................................................................................
