# Supplementary material for: COVID-19 vaccination intention among internally displaced persons in complex humanitarian emergency context, Northeast Nigeria
Source: PLoS One. 2024 Aug 30;19(8):e0308139. doi: 10.1371/journal.pone.0308139 (PMC11364247; doi:10.1371/journal.pone.0308139)
Supplement: S2 File — (PDF) [file pone.0308139.s003.pdf]

S1 File: Assessment algorithm for COVID-19-related knowledge

S/N	Knowledge domains	Anticipated responses	Points*
1	Signs and symptoms of COVID-19¶	Fever	1
		Cough	1
		Fatigue (tiredness)	1
2	Mode of spread of COVID-19	Between people who are in close contact (respiratory droplets/short-range aerosol or short-range airborne transmission)	1
		In poorly ventilated/overcrowded places (long-range aerosol or long-range airborne transmission)	1
		When people touch their mouth, nose, and eyes after touching surfaces and objects contaminated with the virus	1
3	Protective measures against COVID-19	Wearing face mask regularly	1
		Practicing physical distancing	1
		Practicing hand hygiene	1
		Avoiding crowded places	1
		Covering mouth and nose when coughing or sneezing	1
		Vaccination with COVID-19 vaccine	1

* Total of 12 points

¶ One point scored for each of the three most common signs and symptoms of COVID-19
