# Supplementary material for: COVID-19 vaccination intention among internally displaced persons in complex humanitarian emergency context, Northeast Nigeria
Source: PLoS One. 2024 Aug 30;19(8):e0308139. doi: 10.1371/journal.pone.0308139 (PMC11364247; doi:10.1371/journal.pone.0308139)
Supplement: S3 File — (PDF) [file pone.0308139.s004.pdf]

S2 File: List of items used to assess perceived COVID-19 susceptibility, severity, and vaccine effectiveness

S/N	Perception scale	Items[Ψ]
1	Perceived susceptibility to COVID-19	1) I am at risk of getting coronavirus disease (COVID-19)<br>2) It is likely that I will get coronavirus disease (COVID-19)<br>3) I am susceptible to coronavirus disease (COVID-19)
2	Perceived severity of COVID-19	1) Coronavirus disease (COVID-19) is a severe disease<br>2) Coronavirus disease (COVID-19) can have serious consequences on my life and livelihood<br>3) Coronavirus disease (COVID-19) is very harmful
3	Perceived effectiveness of COVID-19 vaccine	1) COVID-19 vaccine protects against serious illness and death from COVID-19<br>2) COVID-19 vaccine is effective in preventing serious COVID-19 disease or COVID-19 death<br>3) If I am vaccinated with COVID-19 vaccine, I will be protected from serious COVID-19 disease or COVID-19 death

[Ψ] Each item was assessed using a 5-point Likert scale ranging from 1 (strongly disagree) to 5 (strongly agree)
