# Supplementary material for: COVID-19 vaccination intention among internally displaced persons in complex humanitarian emergency context, Northeast Nigeria
Source: PLoS One. 2024 Aug 30;19(8):e0308139. doi: 10.1371/journal.pone.0308139 (PMC11364247; doi:10.1371/journal.pone.0308139)
Supplement: Attachment — Submitted filename: Response to Reviewers.docx [file pone.0308139.s006.docx]

**Responses to Academic Editor’s Comments**

**Comment**

The manuscript is well-written and timely, particularly given the invaluable insights garnered from navigating the challenges posed by the COVID-19 pandemic. It is imperative to document such experiences, especially concerning vulnerable populations, to inform future healthcare strategies effectively.

**Response**

We sincerely appreciate the academic editor for these kind comments and inspiring remarks, and for highlighting the public health significance of our research.

**Comment**

However, to provide a comprehensive understanding of the paper's context, it is essential to delineate the impact of the COVID-19 pandemic in Africa and Nigeria, particularly concerning morbidity, mortality, vaccination rates, and other pertinent information. This comparative analysis would elucidate the specific challenges faced by internally displaced persons (IDPs) and set the backdrop for the study's relevance.

**Response**

We acknowledge the insight provided by the academic editor. Accordingly, we have revised the background of the study to reflect the editors’ useful submission and advice (Lines 54 – 68).

**Comment**

While the paper employs various statistical methodologies, it lacks clarity regarding the percentage of IDPs, regardless of their formal or informal status, willing to accept the COVID-19 vaccine.

**Response**

The proportion of internally displaced persons (IDPs) willing to accept COVID-19 vaccine was 63.5% (95% CI: 59.0 – 68.1). We presented this proportion in the result section of the abstract (Lines 36 – 37) and the result section of the body of the manuscript ( Lines 268 – 269). This proportion is a “**weighted proportion”** obtained from the complex sample survey analysis, which was performed to account for the unequal probabilities of selecting participants via a complex sample design rather than a simple random sampling technique. We have elaborated on this pertinent point in the manuscript (Lines 200 – 207).

**Comment**

Additionally, the issue of proximity to health facilities was highlighted, warranting further exploration, particularly in comparison to the general population. Also as COVID 19 is no longer a pandemic, can this also be reflected in the paper.

**Response**

As rightly indicated by the academic editor, the study revealed that IDPs who resided 2 kilometers or farther from the nearest health facility were less likely to accept COVID-19 vaccine. This important finding was extensively discussed in paragraph 7 of the discussion section (Lines 418 – 433). We elaborated on possible explanations for this finding in line with the data from our study. Moreover, we compared this result to the findings of several other studies that reported distance as a major barrier to the uptake of vaccination and other healthcare services at health facilities, even among the general population (Lines 425 – 427). Based on the evidence from our study, which resonates with findings from other similar studies, we highlighted the need for appropriate interventions, including the use of mobile vaccination strategy, to reach and deliver vaccines to IDPs in remote households (Lines 431 – 433). Furthermore, we recognize that COVID-19 is no longer considered a public health emergency; we have reflected this in the manuscript.

**Comment**

Further elaboration on the statistical methodologies employed would enhance the paper's accessibility, especially for readers with limited statistical proficiency. Clarification and additional information on these methodologies would mitigate confusion and facilitate a deeper understanding of the findings.

**Response**

We acknowledge the concern of the academic editor. The comprehensive statistical approaches employed in this study were aimed at enhancing the validity of the study results, particularly because of the complex sample design approach. However, in accordance with the academic editor’s comment, we have provided additional information to enhance the clarity of the statistical methodologies we adopted in the study. Additionally, we have indicated appropriate references for the statistical approaches where necessary (Lines 200 – 221).

**Comment**

Lastly, it is advisable to limit references to publicly available "published work" and consider the feedback provided by Dr. Edina and Bassey. Their insights could further refine and strengthen the manuscript.

**Response**

We appreciate this useful advice. We have limited all our references to only **“published work.”**

**Responses to Reviewers’ Comments
Reviewer 1**

**Comment**

Thank you for the opportunity to review this manuscript which reports on the findings of a complex sample survey analysis conducted to determine the prevalence and factors associated with intention to accept COVID-19 vaccine among internally displaced persons (IDPs) living in Northwest Nigeria. Given the under-representation of IDPs in research studies with this focus and their general exclusion from COVID-19 mitigation programs at the height of the pandemic globally, the findings of this study should make an important contribution to informing equitable and responsive health care provision to this population. The manuscript is well written and presented.

**Response**

We are sincerely grateful to the reviewer for the important remarks and the meticulous review of our paper.

***Major Comments***

**Comment**
To provide further context to the study, the authors should address – as part of the Background section - the burden of COVID-19 in Nigeria (e.g., cases, hospitalization and/or deaths) and provide clarity on the national COVID-19 vaccination program, including access and provision of COVID-19 vaccines among marginalized populations (e.g., IDPs) where possible.

**Response**

We thank the reviewer for this comment and the useful suggestion. Accordingly, we have included this relevant information in the introduction section of the revised manuscript (Lines 54 – 68).

**Comment**
With regards to the Methods, there is a need for further clarity on the types of data collected from the household surveys conducted in July – December 2023 and whether this survey is separate from the semi-structured interviews conducted in the current study. It is unclear from the methods whether the study design adopted was prospective or retrospective. In addition, it is unclear to me when the study activities were conducted (i.e., when were participants recruited and enrolled into this study? What was the duration of this study?).

**Response**

These are pertinent comments. To provide additional clarity on the study design, study participants, and the types of data, we have substantially revised the relevant sub-sections of the “Materials and Methods” section of the manuscript (Lines 130 – 163). Furthermore, the description and categories of study variables are presented in Table 1.

**Comment**
While the comparisons made with findings from general populations from other countries are notable in the Discussion section, the authors are encouraged to also appraise their findings against similar studies conducted among migrant populations (whether IDPs or otherwise) in other parts of the world. For example, you would note from a recently published systematic review and meta-analysis that the findings on vaccine acceptance in this study are comparable, if not higher, than that reported previously among migrant populations globally [Hajissa K, Mutiat HA, Kaabi NA, et al. COVID-19 Vaccine Acceptance and Hesitancy among Migrants, Refugees, and Foreign Workers: A Systematic Review and Meta-Analysis. Vaccines (Basel). 2023;11(6):1070. Published 2023 Jun 6. doi:10.3390/vaccines11061070].

**Response**

We sincerely acknowledge this useful comment. Although there is a dearth of similar studies conducted among internally displaced persons, refugees, and other migrant populations, we have expanded the scope of our discussion to compare the findings of our study with the results of the study mentioned by the reviewer and with the few similar research conducted among refugees and migrant populations in the published literature (Lines 344 – 349).

***Minor Comments***

**Comment**
Line 38, page 2; “…were 18–39 years old, 82.6.0% were females…” Kindly confirm the proportion presented here.

**Response**

We thank the reviewer for this observation. The proportion was **82.6%**; we have corrected this in the manuscript (Line 36).

**Comment**
Line 88, page 5; “Furthermore, a recent, unpublished study conducted among IDPs…” Kindly note the journal guidelines on this and address appropriately: "Do not cite the following sources in the reference list: Unavailable and unpublished work, including manuscripts that have been submitted but not yet accepted (e.g., “unpublished work,” “data not shown”). Instead, include those data as supplementary material or deposit the data in a publicly available database."

**Response**

We noted this comment with gratitude. We have included the citation for the referenced source and reworded the sentence (Lines 98 – 101).

**Comment**
Line 126, page 6; “…and who had not received any dose of COVID-19 vaccine.” Kindly indicate how vaccination history was determined (participant recall or valid vaccination records?).

**Response**

The vaccination status was determined via participant’s recall (or history). We have indicated this in the manuscript (Lines 134 – 136).

**Comment**
Line 127 – 128, page 6; “Data of these participants were extracted from a complex sample, household survey…” For clarity, what type of participant data were extracted from the household survey? I'm struggling to reconcile the household survey conducted in July – December 2023, and the semi-structured interviews described as part of the Data collection and data instrument sub-section. The study is presented here as a prospective one with the retrospective approach only mentioned in the Limitations section. The authors should explicitly state the study design adopted as part of the methods section and avoid ambiguity.

**Response**

We sincerely thank the reviewer for this useful feedback. In line with this suggestion, we have revised the “Materials and Methods” section of the manuscript to provide clarity on the study design, study participants, and data sources ({Lines 130 – 163} and Table 1).

**Comment**
Lines 148 – 149, page 7; “…data for the household survey via face-to-face interviews with study participants…” Where were these interviews conducted. I ask this with relation to the privacy of the interviews conducted.

**Response**

This question is apt. The interviews were conducted within the households to maintain privacy and confidentiality. We have revised the sentence to reflect this information (Lines 147 – 148).

**Comment**
Line 149, page 7; “…using semi-structured data collection instrument.” Kindly provide further clarity on the semi-structured data collection instrument used. If adapted from a publicly available source, please cite appropriately. If not, kindly provide a template as part of supplementary material for ease of reference.

**Response**

We have revised this section of the manuscript. The current study extracted data from a household complex sample survey, which was described extensively in a published article. We have cited this publication in the appropriate section of the revised paper (Lines 131 – 134) and briefly described the sampling methodology and data collection procedure of the household complex sample survey (Lines 140 – 149).

**Comment**
Line 182, page 10; “Nigerian Nigeria (13,300 NGN = 30 US Dollars)” This is unclear to me. Are the authors referring to the Nigerian currency?

**Response**

Yes, NGN is the currency code for Nigerian Naira. We also indicated this in the footnotes of the respective Tables (Lines 171, 259, 278, and 327 ).

**Comment**
Line 237, page 13; “…we obtained verbal informed consent from the participants…” As part of the ethics statement, kindly provide further clarification as to why written informed consent was not possible or required for this study.

**Response**

We acknowledge this important comment. In accordance with the reviewer’s advice, we have provided additional information regarding the informed consent (Lines 233 – 236).

**Comment**
Table 3, page 16; The number of female and male participants in Table 1 and 2 do not correlate. Kindly review all data presented in the manuscript and ensure that they are in fact accurate.

**Response**

We thank the reviewer for bringing this error to our attention. We have made the necessary corrections in Table 3 (Page 16) and double-checked all the data and results in the manuscript for accuracy.

**Comment**
Lines 371 – 372, page 23; “This interpretation suggests that by inducing a higher perception of COVID-19 severity…” With regards to the recommendations made here and later on in the manuscript, I’m wondering if the authors would consider re-framing this to advocate for context-specific and culturally appropriate education campaigns tailored to the needs of IDPs in order to enhance ownership and informed decision-making on COVID-19 vaccination rather than inducing a higher perception of COVID-19 severity.

**Response**

We are grateful for this invaluable suggestion. Accordingly, we have revised the appropriate sections of the manuscript to reflect the reviewer’s advice (Lines 382 – 387 and Lines 455 – 460).

**Comment**
Line 375, page 24; “…communication to shape disease risk reception…” Do the authors mean, "...disease risk perception..."?

**Response**

Yes, we meant to write **“disease risk perception**.” We have corrected this in the paper (Line 385).

**Reviewer 2**

**Comment**
1) The authors needs to re-word the introduction part of the manuscript from line 73 onwards to better reflect the current situation of COVID-19 globally and in Nigeria. The way they have written the introduction portion currently comes across like COVID-19 is still a global pandemic and the world is in lockdown. But COVID-19 pandemic is now properly managed especially in the developing countries. Hence, the authors need to indicate the current COVID-19 situation globally and most importantly indicate the situation in developing countries especially in Nigeria where the study was conducted.

**Response**

We thank the reviewer for these important comments. In line with the reviewer’s advice, we have revised the introduction section of the manuscript to reflect the current COVID-19 situation globally, in Africa, and in Nigeria (Lines 54 – 68).

**Comment**
2) In the study setting, the authors gave a detailed description of the different camps situation aka formal versus informal camps. However, they did not indicate whether the IDPs included and interviewed for the study lived in official or unofficial camps or a mixture of both. The authors should therefore clearly indicate this and include the information in their results section and their study participants and data source section.

**Response**

The study participants included in this study resided in both formal and informal IDPs camps across Borno, Adamawa, and Yobe States (BAY States). We have included this information in the study design and data source sub-section of the manuscript (Lines 134 – 136). Furthermore, the results of COVID-19 vaccination intention (Table 3) and factors associated with intention to accept COVID-19 (Table 5) included data on the status of IDPs camps (formal versus informal).

**Comment**
3) The authors also indicated that 63.5% of IDPs express an intent to accept COVID-19 vaccination but it is not clearly stated how this percentage was calculated or what numbers were used to generate this percentage as the numbers indicated in Table 3 does not equate to 63.5% of IDPs. Thus, the authors need to specify how they obtained the 63.5% indicated in the manuscript. Also, the authors need to indicate the numbers used to obtain this percentage. Furthermore, the authors need to indicate the percentage of IDPs that express an intent to reject the COVID-19 vaccine.
In Table 3, the percentage of each of the features indicated are calculated based on the numbers of IDPs that express and intent to either accept or reject the COVID-19 vaccine. Hence, in the results text description, the authors need to make this clearer as it is not obvious.

**Response**

We acknowledge this important observation. Data of the participants included in the current study were extracted from the database of a household complex sample survey conducted from July to December 2022 in Northeast Nigeria. These participants were recruited via a complex sampling approach rather than a simple random sampling technique. To account for the differential (unequal) probabilities of selecting the participants due to the complex sampling approach, we performed a complex sample survey analysis – the recommended data analysis approach for complex design studies. From this complex survey analysis, we obtained **weighted** statistical estimates, proportions, standard errors, and confidence intervals. In the current study, the **absolute number** of participants who expressed intention to accept COVID-19 vaccine was 1,105 (Line 268). However, the weighted proportion for this figure, obtained from complex sample analysis, was 63.5% (Line 269) instead of the non-weighted proportion of 71.9% expected from a non-complex design analysis. As widely recommended, results of complex surveys (including Demographic and Health Surveys) are usually presented to indicate the **absolute counts** of variables measured together with the **weighted proportions** **or statistical estimates** (with confidence intervals). To improve clarity in our manuscript, we have revised the relevant aspect of the “Data analysis and statistical methods” section of the paper to indicate that the statistical estimates, proportions, and confidence intervals obtained and reported in this study are weighted values (Lines 200 – 216). In addition, we have reworded the results of COVID-19 vaccination intention to reflect both the absolute count and weighted proportions (Lines 268 – 270). In the manuscript, Tables 2, 3, and 4 present the absolute number of participants with weighted proportions (and confidence intervals), while Table 5 shows weighted crude and adjusted odds ratios with confidence intervals.

**Comment**
4) In the discussion section, paragraph 3 contains some redundant information (e.g., misconception, vaccine safety concerns, no felt need, etc.) that had already been stated in paragraph 1. The authors need to consolidate the information in the two paragraphs into one paragraph.

**Response**

We are grateful for this comment. Whereas in Paragraph 1 of the discussion, we summarized the salient findings of the study, paragraph 3 discussed the reasons for intention to reject vaccine. However, in line with the reviewer’s comment, we have expunged redundant information from paragraph 1 and extensively discussed the reasons for intention to reject vaccine in paragraph 3 (Lines 361 – 374).

**Comment**
5) Again, the authors need to indicate whether the perceived COVID-19 severity and vaccine effectiveness were obtained from IDPs living in formal or informal camps

**Response**

We acknowledge this comment. After controlling for co-variates at multivariable logistic regression analysis, IDPs who perceived COVID-19 as a severe illness and those who perceived COVID-19 vaccination as effective, irrespective of whether they resided in formal or informal camps, were significantly more likely to accept the COVID-19 vaccine (Table 5).

**Comment**
6) The authors do not clearly state that over 50% of IDPs express an intent to accept the COVID-19 vaccine but rather they try to belittle this data by giving ambigous percentage of the number of IDPs that expressed an intent to reject the vaccine. They do however, provide a good reference of the percentage of IDPs that expressed an intent to accept the COVID-19 vaccine being below the percentage seen in other generalized populations. The authors are advised to provide clear and unbiased information regarding the percentage value of IDPs that express an intent to accept or reject the COVID-19 vaccine and not play down the fact that over 50% of IDPs did express an intent to take the COVID-19 vaccine.

**Response**

We thank the reviewer for these important comments. In this paper, we reported 63.5% as the weighted proportion of IDPs who expressed intention to accept COVID-19 vaccine. We did not mean to downplay this proportion but rather, we appraised this figure against the results reported in studies conducted among various populations in different countries (Lines 344 – 352). Further, we explained the implications of this result based on facts, figures, and reports documented in the published literature (Lines 352 – 359).

**Comment**
7) When the authors describe the factors affiliated with the IDPs independent decision to take or reject the COVID-19 vaccine, they need to be very clear that the percentage quoted is based on the number of individuals tabulated in each category (e.g., intention to express the COVID-19 vaccine) and not the number of individuals in the total population.

**Response**

We acknowledge this comment. As we stated earlier in our response to comment 3, the proportions and other statistical estimates presented in this study were **“weighted” values** from the complex sample survey analysis. In all the results tables (Tables 2 – 5), we presented absolute numbers (counts) with weighted proportions or statistical estimates (and confidence intervals) in keeping with the recommended approach for presenting the results of complex survey analysis.

**Comment**
8) Finally, the authors need to provide a bit more description about their statistical analyses performed for this study.

**Response**

We appreciate this pertinent comment. Based on this suggestion, we have elaborated on the statistical analysis we performed in this study and provided appropriate references where necessary (Lines 200 – 221).
