# Supplementary material for: COVID-19 vaccination intention among internally displaced persons in complex humanitarian emergency context, Northeast Nigeria
Source: PLoS One. 2024 Aug 30;19(8):e0308139. doi: 10.1371/journal.pone.0308139 (PMC11364247; doi:10.1371/journal.pone.0308139)
Supplement: Attachment — Submitted filename: Response to Reviewers.docx [file pone.0308139.s007.docx]

**Responses to Additional Comments by the Journal and the Reviewers**

**Journal Requirements:**

**Comment**
Please review your reference list to ensure that it is complete and correct. If you have cited papers that have been retracted, please include the rationale for doing so in the manuscript text or remove these references and replace them with relevant current references. Any changes to the reference list should be mentioned in the rebuttal letter that accompanies your revised manuscript. If you need to cite a retracted article, indicate the article’s retracted status in the References list and also include a citation and full reference for the retraction notice.

**Response**

We are grateful for this comment. We have reviewed the reference list for completeness and appropriateness. Accordingly, we have replaced references 2, 6, 10, and 31 in the reference list of the revised manuscript.

**Additional Editor Comments:**
Dear Authors'
Thank you for your thorough revisions and for addressing most of the concerns the reviewers raised. However, there are a few remaining points from Reviewer 2's comments that require your attention for minor revisions.

**Response**

We are grateful for these kind words and happy to address all outstanding reviewers’ comments

**Overall Quality**

**Comment**
The manuscript is well-written, timely, and offers valuable insights into the vaccination intentions of a vulnerable population, which is crucial for informing public health strategies. The statistical analysis is robust and appropriate for the study design.

**Response**

We thank the reviewer for these encouraging remarks.

**Major Comments**

**Comment**
1. Clarification of Study Design and Methods:
• The study design and data collection methods need further clarification. Specifically, there should be a clear distinction between the household surveys conducted and the semi-structured interviews. It is also important to specify whether the study was prospective or retrospective and to include precise dates for participant recruitment and study duration.

**Response**

We appreciate the reviewer for these comments. In line with the reviewer’s suggestion, we have revised the “study design and data source” section of the manuscript to enhance clarity. The current study was a cross-sectional study examining exposure and outcome variables at the same point in time. The precise date of data collection has been included in the revised manuscript.

In the revised manuscript, this section reads in part:

*“This cross-sectional study included a total of 1,537 IDPs aged 18 years and above, spread across 18 formal and informal IDPs camps in Borno, Adamawa, and Yobe States (BAY States) in Nigeria’s northeast region. These IDPs were among the participants enrolled from July 25 to December 5, 2022, in a complex sample household survey to investigate COVID-19 knowledge, risk perception, and adherence to COVID-19 preventive measures among IDPs in the BAY States, Northeast Nigeria. Due to practical and logistic considerations, participants enrolled in the complex sample household survey were sampled using a complex sampling methodology. As of their enrolment in the complex sample household survey, the IDPs included in the current study had not received any dose of COVID-19 vaccine based on history (self-report). The socio-demographic and household data of these 1,537 IDPs, as well as data regarding their COVID-19 knowledge, risk perception, and vaccination intention, were extracted from the database of the complex sample household survey. The sampling methodology and data collection approach for the complex sample household survey have been described elsewhere……* *Data for the complex sample household survey were collected using a semi-structured data collection instrument uploaded on Open Data Kit (ODK). This data instrument is included as Supporting Information S1 File.”* (Lines 133 – 154).

**Comment**

2. Calculation and Presentation of Statistics:
• The authors should provide detailed information on how the 63.5% intention to accept COVID-19 vaccination was calculated. This includes specifying the numbers and methodology used to derive this percentage, as the numbers in Table 3 do not straightforwardly equate to this figure. Additionally, ensure that the percentage of IDPs who intend to reject the vaccine is also clearly stated in the manuscript.

**Response**

We appreciate the reviewer for this comment. The 63.5% intention to accept COVID-19 vaccine was computed using a complex sample data analysis methodology. As we detailed in the first round of reviews, the socio-demographic and household data of the 1,537 participants included in the current study, as well as data regarding their COVID-19 knowledge, risk perception, and vaccination intention, were extracted from the database of a complex sample household survey. Because the participants enrolled in the complex sample household survey were recruited via a complex sampling approach rather than a simple random sampling technique, we performed a complex sample survey analysis to account for the differential (unequal) probabilities of selecting these participants in line with the recommended data analysis approach for complex sampling surveys. Typically, complex sample data analysis yields **weighted statistical estimates, proportions, standard errors, and confidence intervals.**

In the current study, the absolute number of participants who expressed intention to accept COVID-19 vaccine was 1,105 (Line 271). Ordinarily, the non-weighted proportion expected from a non-complex sample design analysis is 1,105/1,537 = 71.9%. However, for the purpose of complex sample analysis, the response of each of the 1,537 participants was adjusted for the survey weight each participant contributed to the analysis based on the sampling probabilities of these participants. The survey weight was obtained by determining the inverse of the sampling probabilities. Each participant's response was thereafter multiplied by the survey weight. For instance, a participant with a higher sampling probability has a lower survey weight than a participant with a lower sampling probability, and vice-versa. In simple terms, each participant’s response was multiplied by the survey weight to obtain a weighted response. The weighted responses of all participants were then summed up and divided by the total population of IDPs to obtain a weighted proportion. This computation was seamlessly executed using the “survey package” of R statistical and computing software, which we utilized for data analysis.

In accordance with the standard statistical procedure for complex sample analysis, we applied the principle of weighted analysis to the weighted logistic regression and other statistical analyses in this study. For the same reason, we used the Rao-Scott chi-square test, a design-adjusted version of the Pearson chi-square test, to determine the relationship between vaccination intention and key participants’ characteristics instead of the conventional Pearson chi-square (Lines 213 – 216). In the revised manuscript, we provided appropriate references for publications that describe the conduct of complex sample data analysis (Line 208 and Line 211).

All the proportions we presented in Table 3 were weighted – the same for all other results we presented in the result tables and elsewhere in the manuscript.

The weighted proportion of participants who intend to reject vaccines was **36.5% (95% CI: 31.9 – 41.0)**. In line with the reviewer's suggestion, we have included this weighted proportion and the 95% confidence interval in the manuscript. This section now reads as follows:

*“Of the 1,537 participants, 1,105 expressed intention to accept COVID-19 vaccine, corresponding to a weighted proportion of 63.5% (95% CI: 59.0 – 68.1), whereas 432 participants, with a weighted proportion of 36.5% (95% CI: 31.9 – 41.0) reported intention to reject COVID-19 vaccine.”*  (Lines 275 – 277).

**Comment**

3. Contextualization of COVID-19 Situation:
• The introduction should be reworded to better reflect the current global and Nigerian context of COVID-19. This should include the current status of COVID-19 as no longer a pandemic and the specific situation in Nigeria, emphasizing how the management of COVID-19 has evolved.

**Response**

We appreciate the reviewer for this comment. In response to a similar comment by the academic editor and Reviewer 1 in the first round of review, we have substantially edited the introduction section of the manuscript to provide appropriate context for our study (Lines 54 – 115). With relevant citations, we provided an elaborate background of the identification, emergence, and situation of COVID-19 globally and in Nigeria. We indicated that the World Health Organization declared an end to COVID-19 global health emergency on May 5, 2023. We described the COVID-19 risk profiles for IDPs and justified the need to optimize COVID-19 vaccination uptake among this population. Citing relevant scientific publications, we noted that although COVID-19 is no longer considered a public health emergency, there is a risk of the emergence of new variants that could be more transmissible and/or more severe with new surges in cases and deaths. Amidst the controversy that trailed the safety and effectiveness of COVID-19 vaccine worldwide, we provided relevant background information regarding the acceptance of COVID-19 vaccine globally, in Nigeria, and among the IDPs in northeast Nigeria. Furthermore, we highlighted the dearth of research on COVID-19 vaccination intention among IDPs in humanitarian emergency contexts to further justify the conduct of our study. We cited publications that asserted that findings of research conducted among different population sub-groups in stable, non-humanitarian contexts cannot simply be extrapolated to inform disease prevention and control interventions in humanitarian emergency situations. We believe the edited introduction of the revised manuscript appropriately contextualized our study, provided essential background information, and highlighted the rationale for the conduct of the study.

**Comment**

4. Comparison with Other Populations:
• The discussion should include comparisons with similar studies conducted among migrant populations, such as refugees and other displaced groups globally. For instance, comparing the findings with those from a recent systematic review and meta-analysis on vaccine acceptance among migrant populations would provide a broader context and relevance.

**Response**

This is a pertinent comment. In line with the reviewer’s suggestion, we have expanded the scope of our discussion to compare our findings with the results of the study mentioned by the reviewer and the results of other similar research conducted among internally displaced persons, refugees, and other migrant populations published in the scientific literature (Lines 354 – 359).

**Minor Comments:**

**Comment**

1. Typographical and Formatting Corrections:
• Line 182: Correct "Nigerian Nigeria" to "Nigerian Naira".

**Response**

We appreciate the reviewer for pointing out this error. We have corrected the error in the revised manuscript (Line 176).

**Comment**

• Line 375: Correct "disease risk reception" to "disease risk perception".

**Response**

We have corrected this in the revised manuscript (Line 395)

**Comment**

2. Additional Details in Methods:
• Provide more details about the semi-structured data collection instrument, including whether it was adapted from an existing source or developed for this study. If adapted, cite the source; if developed, include a template in the supplementary material.

**Response**

The semi-structured data collection instrument was developed by the research team to collect data for the **complex sample household survey** described in the manuscript (Lines 144 – 154). The items in the instrument were informed by a review of the literature on similar subjects. Furthermore, the items were appropriately contextualized to reflect the educational level of the study participants and the peculiarities of the research setting. Since the 1,537 IDPs included in the current study were among the participants enrolled in the complex sample household survey, we have included a template of the data collection instrument for the complex sample household survey in the supplementary materials, in line with the suggestion of the reviewer (Lines 152 – 154).

**Comment**

3. Privacy During Data Collection:
• Indicate where the interviews were conducted, ensuring the privacy and confidentiality of the participants. This is particularly important given the sensitive nature of the information collected.

**Response**

All the participants enrolled in the complex sample household survey, including the 1,537 IDPs in the current study, were interviewed within the households to maintain privacy and confidentiality. We have included this information in the revised manuscript (Lines 150 – 152).

**Specific Revisions Required:**

**Comment**
1. Include Weighted Percentage of IDPs Rejecting Vaccine:
• As noted by the reviewer, state the weighted percentage of IDPs intending to reject the COVID-19 vaccine in the section encompassing lines 280-283, similar to how the intention to accept the vaccine is stated.

**Response**

We appreciate this suggestion. As advised, we have included the weighted percentage of IDPs intending to reject COVID-19 in the result section of the manuscript. This section now reads as follows:

*“Of the 1,537 participants, 1,105 expressed intention to accept COVID-19 vaccine, corresponding to a weighted proportion of 63.5% (95% CI: 59.0 – 68.1), whereas 432 participants, with a weighted proportion of 36.5% (95% CI: 31.9 – 41.0) reported intention to reject COVID-19 vaccine.”*  (Lines 275 – 277).

**Comment**
2. Include Response Text in Methods and Results:
• Incorporate parts of the text from the rebuttal/response to comment 3 of reviewer 2 into the methods section under the data analysis subheading. This should explain the rationale behind the data collection approach and include details on the statistical analysis method. Additionally, include text from the response to comment 5 of reviewer 2 in the appropriate results section.

**Response**

We acknowledge this comment. In line with this suggestion, we have included parts of our initial response to comment 3 of Reviewer 2 and edited the “study design and data sources” section of the revised manuscript to enhance clarity. This section now reads:

*“This cross-sectional study included a total of 1,537 IDPs aged 18 years and above, spread across 18 formal and informal IDPs camps in Borno, Adamawa, and Yobe States (BAY States) in Nigeria’s northeast region. These IDPs were among the participants enrolled from July 25 to December 5, 2022, in a complex sample household survey to investigate COVID-19 knowledge, risk perception, and adherence to COVID-19 preventive measures among IDPs in the BAY States, Northeast Nigeria.* *Due to practical and logistic considerations, participants enrolled in the complex sample household survey were sampled using a complex sampling methodology. As of their enrolment in the complex sample household survey, the IDPs included in the current study had not received any dose of COVID-19 vaccine based on history (self-report). The socio-demographic and household data of these 1,537 IDPs, as well as data regarding their COVID-19 knowledge, risk perception, and vaccination intention, were extracted from the database of the complex sample household survey.”* (Lines 133 – 144).

Furthermore, the details of our statistical analysis approach are provided under the data analysis and statistical methods section of the revised manuscript (Lines 205 – 227). As suggested, we have updated the result section of the manuscript with some of our earlier responses to comment 5 of Reviewer 2. The revised section of the results now reads:

*“Adjusting for co-variates at multivariable logistic regression analysis, IDPs who perceived COVID-19 as a severe illness compared to those who did not (Adjusted Odds Ratio (AOR) = 2.31, [95% CI: 1.35 – 3.96]), and IDPs who perceived COVID-19 vaccine as effective compared to those who did not (AOR = 4.28, [95% CI: 2.46 – 7.44]), irrespective of whether they resided in formal or informal camps, were significantly more likely to accept COVID-19 vaccine.”* (Lines 306 – 311).

**Comment**

3. Correct Currency Terminology:
• Correct the terminology in Line 171 to "Nigerian Naira" and ensure this is consistently used throughout the manuscript.

**Response**

We have noted this error and effected necessary correction in the revised manuscript (Line 176)

**Comment**

Conclusion:
Overall, the manuscript provides significant contributions to understanding COVID-19 vaccination intentions among IDPs in Northeast Nigeria. By addressing the above comments, the authors can enhance the clarity, accuracy, and impact of their study. These revisions will help ensure the manuscript meets the high standards expected for publication.

**Response**

We sincerely appreciate the reviewer for highlighting the public health significance of our study. We have addressed all the reviewers’ comments to enhance clarity and improve the quality of the paper.

**Additional Comments by the Reviewers**
**Reviewer 1**

**Comment**

Thank you for taking the time to carefully consider the comments and suggestions raised during the first round of review. I have the following final issues which need addressing:

Line 182, page 10; “Nigerian Nigeria (13,300 NGN = 30 US Dollars)” This is unclear to
me. Are the authors referring to the Nigerian currency?
Response: Yes, NGN is the currency code for Nigerian Naira. We also indicated this in the
footnotes of the respective Tables (Lines 171, 259, 278, and 327 ).

This has still not been revised to Nigerian Naira as raised during the previous review. Please revise this throughout the manuscript where it appears.

**Response**

We thank the reviewer for this comment. We have corrected this error in the revised manuscript (Line 176)

**Comment**
Lines 394 - 395, page 24; "Although the safety and effectiveness of COVID-19 vaccine are widely disputed..." To avoid this statement being misconstrued, I would encourage the authors to qualify what they mean by "widely disputed". Does this imply that the scientific expert community is uncertain about the safety and effectiveness of all COVID-19 vaccines or does this refer to the uncertainties prominent among the general public?

**Response**

This comment is germane. To provide more clarity and avoid misinterpretation, we have revised the statement to read as follows:

*“Despite the widespread controversy and misinformation regarding the safety and effectiveness of COVID-19 vaccine among the general public, several authors have reported that perceived COVID-19 vaccine effectiveness is a major antidote to vaccine hesitancy.”*

(Lines 404 – 407).

**Reviewer 2**

**Comment**

The authors have greatly revised the manuscript and addressed a large proportion of the raised concerns after reviewing the original manuscript. However, some concerns were not fully addressed and require minor revisions.

The authors did not respond fully to comment (3) of reviewer 2 and should therefore state the weighted % of IDPs that expressed an intent to reject the COVID-19 vaccines in the section encompassing lines 280 - 283 just as they stated for the IDPs who expressed a desire to accept COVID-19 vaccine as indicated in lines 268 - 269 of their revised manuscript. The numbers are already presented in their table so I don't assume it would be difficult to use their statistical analysis method to obtain the requested result.

**Response**

We thank the reviewer for raising this important comment. Accordingly, we have included this weighted proportion and the 95% confidence interval in the revised manuscript (Lines 275 – 277).

**Comment**

Also, the authors need to include some of the text in their rebuttal/response to comment (3) of reviewer 2 in the methods section of the revised manuscript text - specifically the data analysis subheading section. The authors should start by explaining simply why they decided to collect the data for this study using a complex approach as it will provide some context to some of the method description in the data analysis section of their revised manuscript before including where appropriate some of their response to comment 3 as indicated above in the revised manuscript methods data analysis section. Also some of the response text to comment (5) of reviewer 2 should be include in the appropriate results sectionof the revised manuscript text.

**Response**

We acknowledge these comments.

The I,537 IDPs included in the current study were among the participants enrolled in a complex sample household survey conducted during July – December 2022 to investigate COVID-19 knowledge, risk perception, and adherence to COVID-19 preventive measures among IDPs in Borno, Adamawa, and Yobe States (BAY States) in Nigeria’s northeast region. We have revised this section of the manuscript to read as follows:

*“This cross-sectional study included a total of 1,537 IDPs aged 18 years and above, spread across 18 formal and informal IDPs camps in Borno, Adamawa, and Yobe States (BAY States) in Nigeria’s northeast region. These IDPs were among the participants enrolled from July 25 to December 5, 2022, in a complex sample household survey to investigate COVID-19 knowledge, risk perception, and adherence to COVID-19 preventive measures among IDPs in the BAY States, Northeast Nigeria (21). Due to practical and logistic considerations, participants enrolled in the complex sample household survey were sampled using a complex sampling methodology. As of their enrolment in the complex sample household survey, the IDPs included in the current study had not received any dose of COVID-19 vaccine based on history (self-report). The socio-demographic and household data of these 1,537 IDPs, as well as data regarding their COVID-19 knowledge, risk perception, and vaccination intention, were extracted from the database of the complex sample household survey.”* (Lines 133 – 144).

In the revised manuscript, we briefly described the selection of the IDPs camps and the participants for the complex sample household survey (Lines 144 – 152). Additionally, we provided appropriate references for a publication that details the sampling methodology of this complex sample household survey (Line 145).

Similarly, we have revised the data analysis and statistical methods section of the manuscript, as suggested by the reviewer. The section now reads in part:

*“To account for the differential probabilities of selecting study participants using a complex sampling approach rather than a simple random sampling method, we performed a complex sample survey data analysis, consistent with the recommended data analysis approach for complex sampling surveys. We employed an inverse probability weighting approach to determine the survey weight for each study participant. With this survey weight variable, we specified a survey design, created a survey design object, and performed a complex survey analysis to obtain weighted statistical estimates, proportions, standard errors, and confidence intervals. We conducted a weighted univariate analysis to describe participants’ socio-demographics, COVID-19 vaccination intention, and other characteristics. We used the Rao-Scott chi-square test, a design-adjusted version of the Pearson chi-square test, to determine the relationship between vaccination intention and key participants’ characteristics in accordance with the standard statistical procedure for complex sample survey analysis…….”* (Lines 205 – 227)

Furthermore, in line with the reviewer’s advice, we have updated the result section of the manuscript with some of our earlier responses to comment 5 of Reviewer 2 to read as follows:

*“Adjusting for co-variates at multivariable logistic regression analysis, IDPs who perceived COVID-19 as a severe illness compared to those who did not (Adjusted Odds Ratio (AOR) = 2.31, [95% CI: 1.35 – 3.96]), and IDPs who perceived COVID-19 vaccine as effective compared to those who did not (AOR = 4.28, [95% CI: 2.46 – 7.44]), irrespective of whether they resided in formal or informal camps, were significantly more likely to accept COVID-19 vaccine.”* (Lines 306 – 311).

**Comment**
Finally, the authors need to correct the error in Line 171 Nigerian Nigeria to Nigerian Naira as indicated in lines 259, 278 and 327.

**Response**

We are grateful for this comment. We have corrected this error in the revised manuscript (Line 176).
